# Large-Scale Clonal Analysis Resolves Aging of the Mouse Hematopoietic Stem Cell Compartment

**DOI:** 10.1016/j.stem.2018.03.013

**Published:** 2018-04-05

**Authors:** Ryo Yamamoto, Adam C. Wilkinson, Jun Ooehara, Xun Lan, Chen-Yi Lai, Yusuke Nakauchi, Jonathan K. Pritchard, Hiromitsu Nakauchi

**Affiliations:** 1 Institute for Stem Cell Biology and Regenerative Medicine, Stanford University School of Medicine, Lorry I. Lokey Stem Cell Research Building, 265 Campus Drive, Stanford, CA, USA; 2 Department of Genetics, Stanford University, Stanford, CA, USA; 3 Division of Stem Cell Therapy, Center for Stem Cell Biology and Regeneration Medicine, Institute of Medical Science, University of Tokyo, Tokyo 108-8639, Japan; 4 Department of Biology, Stanford University, Stanford, CA, USA; 5 Howard Hughes Medical Institute, Stanford University, Stanford, CA, USA

**Keywords:** hematopoietic stem cell, hematopoiesis, HSC, aging, stem cell aging, single cell, clonal analysis, multipotency, self-renewal

## Abstract

Aging is linked to functional deterioration and hematological diseases. The hematopoietic system is maintained by hematopoietic stem cells (HSCs), and dysfunction within the HSC compartment is thought to be a key mechanism underlying age-related hematopoietic perturbations. Using single-cell transplantation assays with five blood-lineage analysis, we previously identified myeloid-restricted repopulating progenitors (MyRPs) within the phenotypic HSC compartment in young mice. Here, we determined the age-related functional changes to the HSC compartment using over 400 single-cell transplantation assays. Notably, MyRP frequency increased dramatically with age, while multipotent HSCs expanded modestly within the bone marrow. We also identified a subset of functional cells that were myeloid restricted in primary recipients but displayed multipotent (five blood-lineage) output in secondary recipients. We have termed this cell type latent-HSCs, which appear exclusive to the aged HSC compartment. These results question the traditional dogma of HSC aging and our current approaches to assay and define HSCs.

## Highlights

• Single-cell transplantation reveals dramatic age-related changes in HSC composition
• MyRPs/MySCs increase with age as a frequency of whole BM cells and the HSC compartment
• Latent-HSCs were identified exclusively in the aged bone marrow
• Latent-HSCs have restricted potential in primary, but not secondary, transplants

## Graphical Abstract

**Figure:**
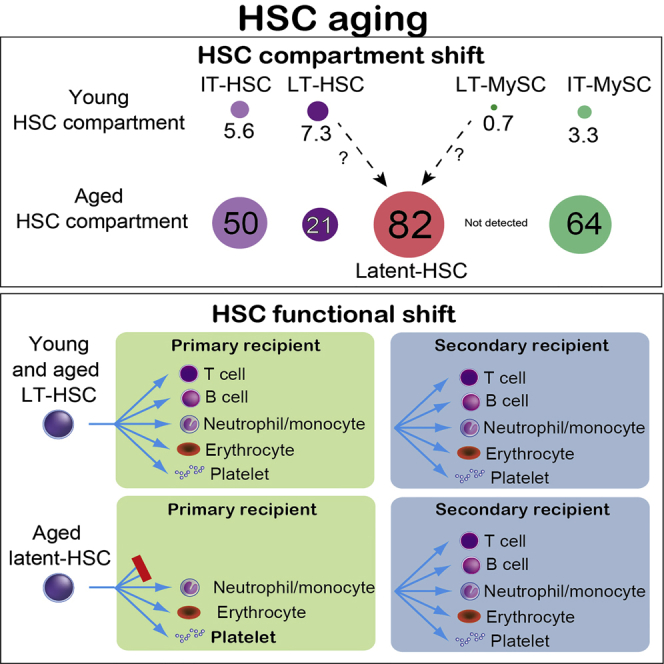

## Teaser

Yamamoto et al. explore age-related changes to HSC function through large-scale clonal analysis using single-cell transplantation. They find large increases in myeloid-restricted repopulating progenitors (MyRPs) as well as a population of MyRPs that display a broader differentiation capacity only in secondary transplants, suggesting additional mechanisms contributing to hematopoietic aging.

## Introduction

Long-term functionally multipotent mouse hematopoietic stem cells (HSCs), as defined by engraftment following primary and secondary transplantation, are found within a phenotypic CD34^−/low^Flt3^−^c-Kit^+^Sca-1^+^Lineage^−^ (CD34^−^KSL) bone marrow (BM) cell population (Osawa et al., 1996). Functional HSCs can be further enriched using CD150 expression (Kiel et al., 2005, Morita et al., 2010). However, even the most phenotypically “pure” HSC population remains functionally heterogeneous in terms of lineage output and self-renewal capacity (Wilson et al., 2015). For example, HSCs display heterogeneity in blood reconstitution duration and are commonly resolved into short-term (ST), intermediate-term (IT), and long-term (LT) repopulating HSCs (Yamamoto et al., 2013).

HSC multipotency has been traditionally defined by neutrophil/monocyte (nm) and B/T lymphocyte (B/T) differentiation. However, without considering the contribution to erythrocytes (E) and platelets (P), such definitions only partially describe HSC multipotency. As the two most abundant and essential blood cell components, understanding E and P lineage contribution is also of significance in the development of strategies to improve clinical BM transplantation. Using clonal analysis in combination with five-blood lineage (nm, B, T, E and P) analysis, we previously determined the functional heterogeneity of the phenotypic HSC (pHSC) compartment in young mice (Yamamoto et al., 2013). In doing so, we identified a subset of pHSCs that were functionally myeloid-restricted repopulating progenitors (MyRPs). Using paired-daughter cell analysis, we further demonstrated MyRPs could be directly generated from HSCs via a myeloid-bypass pathway through a single-cell division event.

While the majority of MyRPs in young mice were ST/IT-MyRPs, a minor population was LT-MyRPs (engrafting in secondary recipients), suggesting that MyRPs may resolve into distinct subpopulations. Further evidence for this comes from an independent analysis of HSCs at five-blood-lineage resolution (Carrelha et al., 2018). Through using a *Vwf-mCherry* reporter mouse line, Carrelha et al. identified a population of potently self-renewing HSCs within the CD150^+^CD34^−^KSL population that had myeloid and lymphoid capacity (in the context of *in vitro* differentiation assays) but displayed P-restricted output *in vivo* (in primary and secondary transplantation assays). In young mice, this population of P-restricted HSCs appeared to be a minor subset of the phenotypic CD150^+^CD34^−^KSL population (just ∼2%). According to our previously defined criteria, these P-restricted HSCs would be LT-MyRPs, which we observed at similar frequencies within our own transplantation assays (Table S1). These data suggest that ST-MyRPs and LT-MyRPs must be considered as distinct populations within the pHSC compartment.

Native hematopoiesis has also recently been investigated at five-blood-lineage resolution (Rodriguez-Fraticelli et al., 2018). Through elegant transposon-based barcoding experiments, Rodriguez-Fraticelli et al. found that pHSCs were a major source of the megakaryocyte/P lineage. These data are highly consistent with the presence of MyRPs and activity of the myeloid-bypass pathway in native hematopoiesis. Further evidence for direct differentiation of HSCs into MyRPs came from HSC cell-division counting experiments by Bernitz et al., which suggested that MyRP-like cells were generated from LT-HSCs after four symmetric self-renewal cell division events (Bernitz et al., 2016).

Dysfunction within the HSC compartment is thought to be a key mechanism underlying age-related hematopoietic perturbations (Elias et al., 2017). Aged HSCs are reported to show altered self-renewal (Beerman et al., 2010, Dykstra et al., 2011, Sudo et al., 2000), impaired homing and engraftment upon transplantation (Dykstra et al., 2011), myeloid-biased differentiation (Dykstra et al., 2011, Sudo et al., 2000), P-biased differentiation (Grover et al., 2016), and megakaryocytic/erythroid-biased gene expression patterns (Rundberg Nilsson et al., 2016). However, most of these observations have been made using population-based methods using only three- (or four)-lineage analysis. Here, we have defined how the pHSC compartment changes during aging at five-blood-lineage resolution. From over 400 clonal transplantation experiments, we demonstrate there is a large increase in MyRP frequency with age. A modest increase in the frequency of functional HSCs within the BM was also observed. Unexpectedly, we also identified a subset of functional cells within the aged pHSC compartment that generated only myeloid (P, E, and/or nm) cells in primary recipients but displayed multipotent (P, E, nm, T, and B) output in secondary recipients. We termed this age-specific functional cell type latent-HSCs. Our clonal analysis of HSC aging therefore questions the current dogma of HSC compartment aging and current approaches to define HSC function.

## Results

### Aging Is Associated with Altered Functional HSC Composition and an Expanded MyRP Population

**Figure 1:**
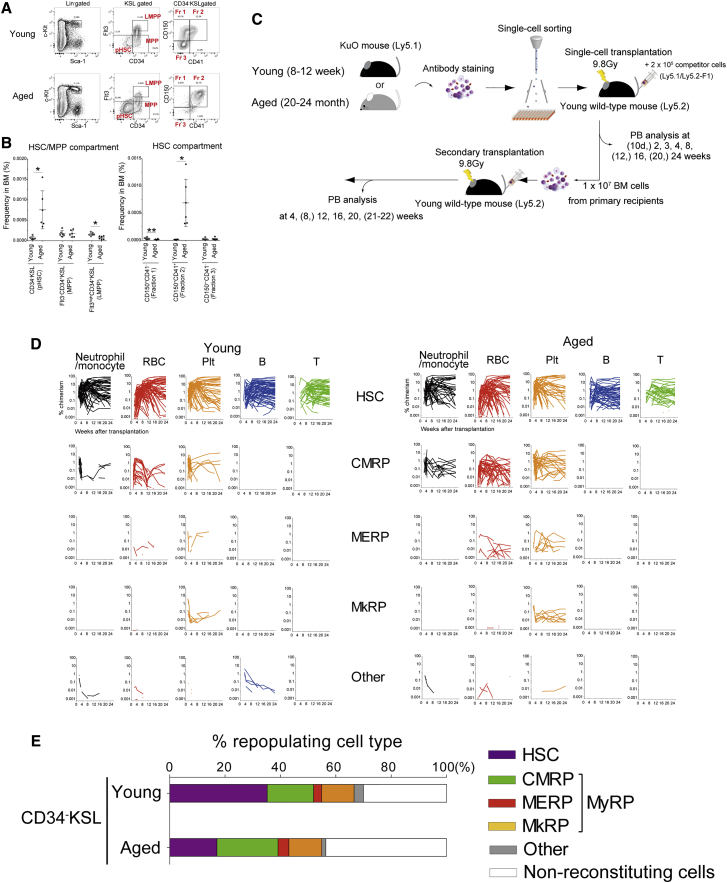
The Phenotypic HSC Compartment Changes with Age (A) Representative flow cytometric data of young and aged bone marrow (BM): MPP, multipotent progenitor; LMPP, lymphoid-primed multipotent progenitor; Fr 1, fraction 1; Fr 2, fraction 2; Fr 3, fraction 3. (B) Frequency of the HSC/MPP population (left) and HSC subpopulations (right) in young and aged BM (as detailed in A). Dots represent individual mice, and horizontal lines indicate median ± SD. (C) Summary of primary and secondary transplantation experiments to test potential of young and aged single phenotypic HSCs. Single CD34^−^KSL, fraction 1, fraction 2, or fraction 3 cells were sorted from BM cells of Kusabira Orange (KuO) mice and were individually transplanted with 2 × 10^5^ BM competitor cells from Ly5.1/Ly5.2-F1 mice into lethally irradiated Ly5.2 mice. Chimerism of KuO^+^ neutrophils/monocytes, erythrocytes, platelets, B cells, and T cells in peripheral blood (PB) was analyzed at 2, 3, 4, 8, (12), 16, (20), and 24 weeks after primary transplantation. Secondary transplantation assays were performed by transferring 1 × 10^7^ whole BM cells from primary recipient mice. PB chimerism was analyzed 4, 12, 16, 20, (and 21–22) weeks in secondary recipients. (D) PB chimerism of individual single young and aged HSCs in a total of 421 primary recipients (as described in C), separated based on lineage output. (E) Estimated frequency of functional HSCs, CMRPs, MERPs, MkRPs, and “other” within the young and aged pHSC compartment, derived from single-cell transplantation assays (Table 1). CMRPs, MERPs, and MkRPs are subsets of MyRPs. “Non-reconstituting” denotes no PB reconstitution of KuO^+^ cells in primary recipients.

To directly compare HSC heterogeneity during aging, it was first important to define pHSCs regardless of age. Young and aged functional HSCs are reportedly enriched in the CD150^+^CD48^−^ gate of the CD34^−^KSL population (Yilmaz et al., 2006). To purify HSCs, we used Sca-1^high^ cells within the KSL population, since Sca-1^low^ cells do not contain functional HSCs (Wilson et al., 2015). With this HSC gating strategy, 97% of the (CD34^−^KSL) HSC compartment in young (8- to 12-week-old) and aged (20- to 24-month-old) mice were negative for CD48 (Figure S1A). These data suggested that CD48 staining was not essential to purify functional HSCs both in young and aged mice. Consistent with previous studies (Sudo et al., 2000), the BM frequency of the pHSC (CD34^−^KSL) compartment increased ∼10-fold in aged mice (Figures 1A and 1B).

We previously resolved the CD150^+^CD34^−^KSL pHSCs population in young mice into two fractions based on CD41 expression: fraction 1 (CD150^+^CD41^−^CD34^−^KSL) cells were enriched for functionally multipotent LT/IT-reconstituting HSCs, whereas fraction 2 (CD150^+^CD41^+^CD34^−^KSL) cells tended to show a myeloid-committed phenotype. By contrast, CD150^−^CD41^−^CD34^−^KSL (termed fraction 3) cells were predominantly ST-reconstituting HSCs. Within the pHSC compartment, the frequency of CD150^+^CD41^−^CD34^−^KSL (fraction 1) cells, in which most young functional HSCs are found (Yamamoto et al., 2013), was significantly decreased in aged mice. By contrast, the frequency of CD150^+^CD41^+^CD34^−^KSL (fraction 2) was dramatically increased (20-fold) (Figures 1A and 1B), as previously reported (Gekas and Graf, 2013). Smaller changes to phenotypic hematopoietic progenitor populations were also observed (Figure S1B), in line with previous reports (Elias et al., 2017).

To directly interrogate the functional characteristics of individual young and aged CD34^−^KSL cells, we performed single-cell transplantation into lethally irradiated young mice. We initially transplanted single CD34^−^KSL cells from young (8- to 12-week-old) and aged (20- to 24-month-old) Ly5.1 Kusabira Orange (KuO) mice (Hamanaka et al., 2013) into a total of 76 lethally irradiated young Ly5.2 mice, together with 2 × 10^5^ competitor cells from Ly5.1/Ly5.2-F1 mice (Figure 1C). As we observed significant changes in CD150 and CD41 expression within the CD34^−^KSL population with age, we also transplanted single CD150^+^CD41^−^CD34^−^KSL (fraction 1), CD150^+^CD41^+^CD34^−^KSL (fraction 2), and CD150^−^CD41^−^CD34^−^KSL (fraction 3) cells from young and aged mice into a total of 370 lethally irradiated Ly5.2 young mice.

We defined the 421 donor cells according to their contribution to the five peripheral blood (PB) lineages (nm, E, P, B, T) and their duration of reconstitution over 24 weeks in primary recipients, as previously defined (Yamamoto et al., 2013). Reconstituting cells were classified into HSC, MyRP (common myeloid repopulating progenitors [CMRP], megakaryocyte-erythroid repopulating progenitors [MERP], and megakaryocyte repopulating progenitor [MkRP]), and ”others” (reconstituting cells that did not meet HSC- or MyRP-type reconstitution criteria) (Figure S1D). To avoid missing functional cells, we set our threshold at 0.005%. However, similar results were seen if a threshold of 0.1% was used instead (Figures S1E and S1F). By combining all single-cell transplantation datasets with the frequency of fractions 1–3 in young and aged mice, we calculated the frequency of these subsets within the pHSC compartment (Figure 1E) and in total BM (Table S2). In terms of frequency within the pHSC population, HSCs decreased by half with age, MyRPs (including CMRPs, MERPs, and MkRPs) were found at similar frequencies, and undetectable cells (termed non-reconstituting cells) increased (Figure 1E). Functional composition was significantly different between young and aged pHSCs (Figure S1G; chi-square test; p < 0.05). However, when considered as a frequency of total BM cells, HSCs increased 5.2-fold, while MyRPs expanded 13-fold (Table S2).

### LT/IT Myeloid-Restricted Repopulating Cells Expand 16-Fold with Age, Suggesting Potent Self-Renewal Capacity

**Figure 2:**
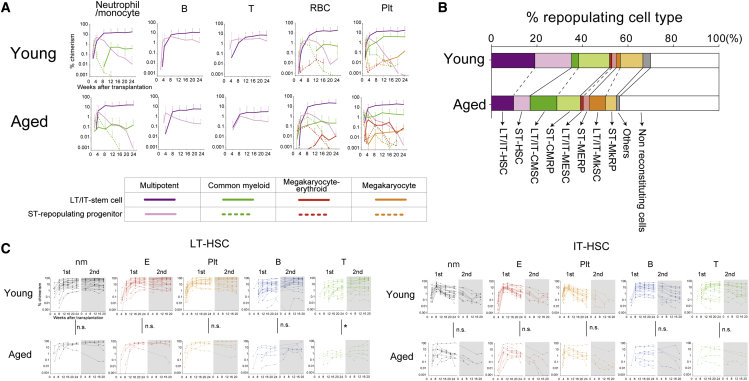
Functional Comparison of Young and Aged HSCs by Single-Cell Transplantation (A) Average chimerism of young and aged HSCs and MyRP/MySC subsets within primary recipients over 24 weeks. Functional cell types subdivided based on reconstitution duration into long-term (LT) or intermediate-term (IT) and short-term (ST) RCs. Data points indicate mean ± SD. (B) Estimated frequency of each functional cell type (HSCs, CMRPs/CMSCs, MERPs/MESCs, MkRPs/MkSCs, and “other”) divided into LT/IT- and ST-repopulating subsets within the young and aged pHSC compartment, derived from single-cell transplantation assays (Table 1). (C) PB chimerism of individual single young and aged LT- and IT-HSCs in primary and secondary recipients. Blood lineage chimerism in primary recipients at 24 weeks was compared using an unpaired t test (^∗^p < 0.05).

We next focused on the reconstitution kinetics of each repopulating cell type in primary recipients (Figure 2A). Repopulating cells (RCs) were classified based on duration of reconstitution capacity and designated as ST or LT/IT cells, as previously defined (Yamamoto et al., 2013). As we had seen before, most LT/IT-RCs in the young pHSC compartment were functional HSCs (Figure 2B). However, in the aged pHSC compartment, more than half of the LT/IT-RCs lacked lymphoid potential (Figure 2B), corresponding with the myeloid-biased reconstitution by aged BM cells. Strikingly, LT/IT-MyRPs expanded 16-fold within the BM with age, while ST-MyRPs expanded by a more modest 7.5-fold with age (Table S3).

The dramatic increase in the frequency of LT/IT-MyRPs suggests that the P-restricted (and myeloid-restricted) HSCs described by Carrelha et al. (2018) likely expand with age. This is consistent with their suggestion that this population has potent self-renewal activity. Based on these data, we suggest renaming LT/IT-MyRPs. To distinguish them from multipotent LT-HSCs, ST-MyRPs, and progenitors (e.g., megakaryocyte progenitors [MkP], megakaryocyte-erythroid progenitors [MEPs], and common myeloid progenitors [CMPs]), we suggest naming them myeloid-restricted stem cells (MySCs), which includes megakaryocyte stem cells (MkSCs), megakaryocyte-erythroid stem cells (MESCs), and common myeloid stem cells (CMSCs) (see Figure S2 for a glossary of functional cell terminology).

### Multipotent LT-HSCs Expand 3-Fold with Age but Display Reduced T-Lymphoid Potential and Limited Self-Renewal Capacity at the Clonal Level

To further resolve LT- and IT-RCs, we performed secondary transplantation assays. While it was not possible to perform secondary transplantation assays on all primary recipients, we transplanted BM from 89 primary recipients (48 with young pHSCs and 41 with aged pHSCs) into a total of 153 secondary recipients (Figure 2C). We initially considered multipotent HSC reconstitution in secondary recipients. While nm, E, P, and B engraftment were comparable between young and aged LT-HSCs, T lymphoid production was significantly decreased from aged LT-HSCs in primary and secondary recipients (Figure 2C). These data confirm that even at the level of single LT-HSCs, T-potential is reduced with age. However, it is worth noting that the loss of T output was not seen in aged IT-HSCs (as compared to young IT-HSCs).

Although LT-HSCs decreased as a frequency of the pHSC compartment, we estimate the frequency within the BM increases with age 2.9-fold, from 7.3 to 21 cells per 10^6^ BM cells (Table 1). These data suggest that multipotent LT-HSCs display modest self-renewal capacity. To more directly quantify self-renewal of young LT-HSCs, we set up single-cell secondary transplantation assays from primary recipient mice displaying a functional LT-HSC phenotype following transplantation of single CD150^+^CD41^−^CD34^−^KSL (fraction 1) cells (Figure S3A). As in our primary single-cell transplantation assays, we transplanted single CD150^+^CD41^−^CD34^−^KSL (fraction 1), CD150^+^CD41^+^CD34^−^KSL (fraction 2), and/or CD150^−^CD41^−^CD34^−^KSL (fraction 3) cells from the primary recipients into a total of 92 mice.

**Table 1:** Frequencies of Each Cell Type among Nucleated BM Cells from Secondary Transplant Assay

Functional Cell Type	Young	Aged	Fold Increase
LT-HSC	7.3	21.0	2.9
IT-HSC	5.6	49.9	9.0
ST-HSC	10.8	52.4	4.9
LT-latent-HSC	0.0	82.0	n/a
LT-CMSC	0.3	0.0	n/a
IT-CMSC	1.7	34.2	19.7
ST-CMRP	9.2	75.1	8.2
LT-MESC	0.3	0.0	n/a
IT-MESC	0.3	9.1	27.8
ST-MERP	1.3	19.1	14.7
LT-MkSC	0.0	0.0	n/a
IT-MkSC	1.2	20.4	17.1
ST-MkRP	6.7	35.1	5.2
Other	2.3	10.8	4.7
Non-reconstituting cells	20.3	316.0	15.6

Data presented as the estimated number of each cell type per 10^6^ BM cells and fold increase from young to aged BM. Frequencies of each cell type were estimated using results of single-cell transplantation assays (including our previously reported data; Yamamoto et al., 2013) and frequencies of fractions 1, 2, and 3 (Figure 1). Latent-HSCs show LT/IT-MyRP-type reconstitution in primary recipients. Frequencies were calculated from a total of 245 young HSC transplants and 196 aged HSC transplants (excluding dead mice). From these, 94 and 36 were used for secondary transplantation, respectively. CMSC, common myeloid stem cell; IT, intermediate-term; LT, long-term; MESC, megakaryocyte-erythroid stem cell; MkSC, megakaryocyte stem cell; n/a, not applicable; ST, short-term. “Other” denotes reconstituting cells that did not fit the criteria of the above functional cell types.

Consistent with our previous findings, within the primary recipient, functional HSCs and MyRPs were found within fraction 1, but MyRPs were the major cell type (59.5%) and HSCs were a more minor population (19%) (Figure S3B). Notably, all MyRPs displayed ST-repopulating kinetics. Fraction 3 also contained both functional HSCs and MyRPs (22.2% and 3.7%, respectively). By contrast, the only functional fraction 2 cells were MyRPs (48%) (Figure S3C). These data suggest that single LT-HSCs can re-establish functional heterogeneity within the pHSC compartment following transplantation but with a bias toward ST-MyRP generation.

### LT-RCs from the Aged BM Display a Latent-HSC Phenotype

**Figure 3:**
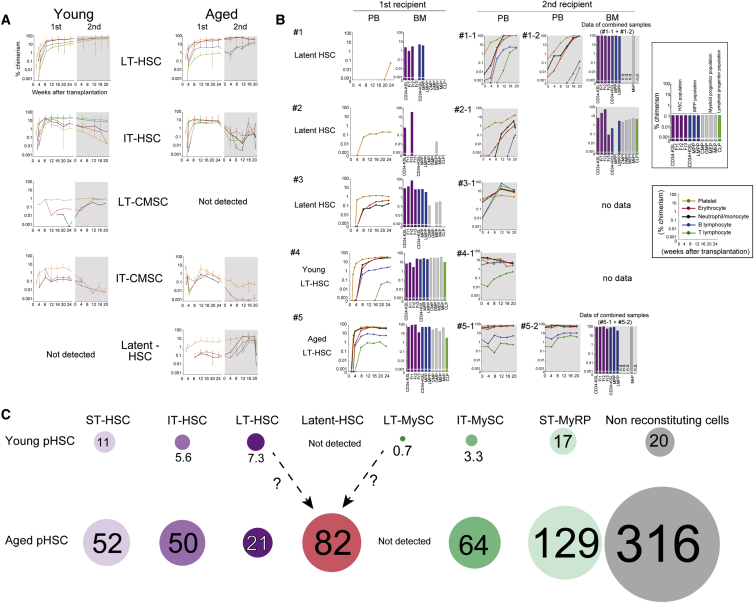
A Subset of Aged MyRPs Display a Latent-HSC Phenotype (A) Average chimerism kinetics of young and aged LT-HSCs (n = 24 and 6, respectively), IT-HSCs (n = 24 and 10, respectively), LT-CMSCs (n = 1 and 0, respectively), IT-CMSCs (n = 7 and 3, respectively), and latent-HSCs (n = 0 and 7, respectively) using all single-cell transplantation datasets (including our data published in Yamamoto et al., 2013). Each line represents the frequency of donor-derived cells in the blood of a single recipient after primary transplantation and secondary transplantation. (B) Representative PB and BM chimerism in primary and secondary recipients for latent-HSCs (1–3), young LT-HSCs (4), and aged LT-HSCs (5). The frequency of KuO^+^ phenotypic stem and progenitor cells within the BM was determined at 24 weeks after primary transplantation and 20 weeks after secondary transplantation. Chimerism of KuO^+^ phenotypic HSCs, fraction 1, fraction 2, fraction 3 (highlighted in purple), MPPs, LMPPs (highlighted in blue), CMPs, GMPs, MEPs, MkPs (highlighted in gray), or CLPs (highlighted in green) is show in the bar graph. n.d. denotes no data. BM cells from mouse 1-1 and 1-2 and mouse 5-1 and 5-2 were pooled and analyzed. (C) Schematic of age-related changes to the mouse HSC compartment. Circle size/number represents the frequency of each cell type per 10^6^ BM cells. With age, the pHSC compartment expands ∼10-fold, largely due to a large increase in MyRPs/MySCs and non-reconstituting cells. Absolute numbers of functional HSCs (fHSCs) only increases modestly and therefore become less frequent within the pHSC compartment. Cumulatively, this leads to the reduced function of the HSC compartment including loss of lymphoid (B and T cell) lineage output. However, the aged pHSC compartment also contains latent HSCs, which display myeloid-restricted output in primary recipients but multipotent (five blood-lineage) output in secondary recipients. We have not detected latent-HSCs in the young pHSC compartment.

To further interrogate the LT activities of MyRPs during aging, we assessed primary recipients with MyRP-type reconstitution in secondary transplantation assays. Surprisingly, aged MyRPs exhibited a phenotypic change in PB lineage output in secondary recipients; LT-RCs with myeloid-restricted reconstitution in primary recipients acquired lymphoid potential (B and/or T) in secondary recipients (Figures 3A, 3B, and S4A, and S4B). This was seen in seven of the ten primary recipients displaying a MyRP-type reconstitution, with the remaining three not displaying LT reconstitution in secondary recipients. Strikingly, two mice with a P-restricted reconstitution pattern in the primary recipients displayed all five-blood-lineage output in secondary recipients (Figure 3B). We have termed this functional population latent-HSCs, a subpopulation of functionally multipotent HSCs that only display differentiation into a limited subset of lineage potentials during 24 weeks in primary recipients. All latent-HSCs identified in this study were from the aged CD150^+^CD41^+^CD34^−^KSL (fraction 2) population. Such a phenotype was never detected in mice transplanted with young pHSCs, both in this study and in our previous study (a total of 409 young single pHSC transplants). By contrast, LT-repopulating MySCs were detected in young BM at low frequencies (Table 1) but were not identified in aged BM.

To investigate the latent-HSC phenotype further, we compared latent-HSC BM chimerism in primary and secondary recipients with that of other HSC types (Figures 3B and S4B). Primary recipients of latent-HSCs as well as young and aged LT-HSCs contained CD34^−^KSL cells within the BM, which suggest that transplanted single latent-HSCs have self-renewal activity. However, immunophenotypically defined (not functionally defined) downstream hematopoietic progenitor cell (HPC) populations had significantly (p < 0.01) lower, or no detectable, donor chimerism (Figures 3B, S4B, and S4C). Chimerism within these HPC populations increased in the BM of secondary recipients displaying a latent-HSC phenotype. This is compatible with the output of mature blood (lymphoid) lineages in these secondary recipients. By contrast, young and aged LT-HSCs usually reconstitute BM stem and progenitor fractions in both primary and secondary recipients to the same extent (Figures 3B and S4). These data suggest that latent-HSCs proliferate (via self-renewal that expands the CD150^+^CD41^+^CD34^−^KSL population) but largely fail to differentiate, particularly along the lymphoid lineage, within in primary recipients. However, the mechanism underlying the latent-HSC phenotype requires further clarification in future studies.

## Discussion

Here, we performed large-scale single-cell transplantation assays to investigate functional HSC heterogeneity during aging at five-blood-lineage resolution. Consistent with previous studies (Dykstra et al., 2011, Sudo et al., 2000), we observed a 10-fold increase in the BM frequency of CD34^−^KSL pHSCs in aged C57BL/6 mice. We found that functional LT-HSCs also expand in the BM with age, although by a more modest 2.9-fold (Table 1; Figure 3C). Similar increases were seen in the frequency of total functional HSCs (including ST-, IT-, and LT-HSCs). However, because we did not directly compete single young HSCs against single aged HSCs, we cannot draw conclusions about age-related changes to the potency of HSCs (i.e., level of donor chimerism). Several direct comparisons using bulk HSC transplantations assays have suggested that young HSCs outcompete aged HSCs (Elias et al., 2017). Such an experiment is difficult using our current methodology but could be achieved by combining our approaches with *in vivo* cellular barcoding technology (Rodriguez-Fraticelli et al., 2018).

Although absolute numbers of functional HSCs increased in the BM with age, functionally multipotent HSCs become less frequent within the pHSC compartment due to a large increase in MyRPs/MySCs. Besides the 13-fold increase in MyRPs/MySCs (all reconstituting types in primary recipients) within the aged BM, non-reconstituting pHSCs also increased nearly 16-fold with age (Table 1; Figure 3C), which combined are responsible for the “dilution” of functional HSCs within the pHSC compartment. Through clonal analysis of HSC aging, we were able to distinguish the expansion of myeloid-restricted MyRPs from the myeloid bias of multipotent HSCs.

Accumulation of MyRPs/MySCs with age suggests the possibility that the myeloid-bypass pathway (Yamamoto et al., 2013) is the major route of HSC differentiation. Correspondingly, our single-cell secondary transplantation assays (Figure S3) suggested that ST-MyRP accumulation may at least in part be driven by cell division of LT-HSCs. Our analysis also highlights that while the frequency and composition of the pHSC compartment changes dramatically with age, the frequency of mature cell types within the hematopoietic system only changes gradually (Figure S1C). This disconnect between HSC/MyRP frequencies and mature blood cell frequencies is likely due to still poorly defined tissue homeostatic mechanisms. Interestingly, “clonal hematopoiesis”, the clonal dominance of certain somatically acquired clonal mutations in human PB, has recently been strongly correlated with age and is now considered to be a precursor (or “pre-leukemic”) state to hematological malignancies (Genovese et al., 2014, Jaiswal et al., 2014, Jan et al., 2012, Shlush et al., 2014). Our data suggest that we must consider as potential origins of clonal hematopoiesis not only HSCs but also self-renewing MyRPs/MySCs.

One of the most surprising findings from our single-cell transplantation assays was the identification of latent-HSCs, a subpopulation of aged pHSCs that displayed myeloid-restricted output over 24 weeks in primary recipients but then displayed a five blood-lineage HSC phenotype following transplantation into secondary recipients. This delay in the functional output of latent-HSCs suggests that we may need to re-evaluate current methods to assay HSC function. Surprisingly, latent-HSCs were more frequent within the aged BM than LT-HSCs, found at ∼82 per 10^6^ BM cells (compared to 21 per 10^6^ BM cells for LT-HSCs) (Figure 3C). A better understanding of the latent-HSC phenotype could help to develop therapeutic strategies to “rebalance” hematopoiesis in aged individuals. An important next step for this will be the development of methods to prospectively isolate latent HSCs for molecular analysis.

While the mechanistic basis of the latent-HSC phenomenon remains to be determined, several hypotheses are apparent. First, the weak differentiation output is reminiscent of the recently described activity of native HSCs (Sun et al., 2014), suggesting primary transplantation stress may be insufficient to induce differentiation and hematopoietic system reconstitution. Latent-HSCs may have multipotent potential in primary recipients but only display a limited subset of their potential in PB cell output. Second, the repression of lymphoid output by MySCs described by Carrelha et al. (2018) may be eroded with age and could result in multipotent output in secondary recipients. Genetic and epigenetic alterations are closely correlated with phenotypic and functional changes to HSCs and could be responsible. Third, our transplantation assays used young mice as recipients, so it is possible that transfer of aged cells into a young BM microenvironment could lead to “rejuvenation” or “conversion” of MySCs into transplantable multipotent HSCs through non-cell-autonomous mechanisms. Further experimentation is warranted to test such hypotheses and determine the physiological relevance of latent-HSCs. Nonetheless, the existence of latent-HSCs in aged mice not only leads us to reconsider the HSC definitions and assay systems but also opens up a new paradigm in HSC biology.

## STAR★Methods

### Key Resources Table

**Table:** 

REAGENT or RESOURCE	SOURCE	IDENTIFIER
**Antibodies**		

Biotin anti-CD4	eBioscience	Cat#13-0041-85, RRID:AB_466326
Biotin anti-CD8	eBioscience	Cat# 13-0081-86, RRID:AB_466348)
Biotin anti-B220/CD45RA	eBioscience	Cat# 36-0452-85, RRID:AB_469753
Biotin anti-TER-119	eBioscience	Cat# 13-5921-85, RRID:AB_466798
Biotin anti-Ly-6G/Ly-6C (RB6-8C5)	eBioscience	Cat# 13-5931-85, RRID:AB_466801
Biotin anti-CD127 (A7R34)	eBioscience	Cat# 13-1271-85, RRID:AB_466589
Biotin anti-CD3e Monoclonal Antibody (145-2C11)	eBioscience	Cat# 13-0031-85, RRID:AB_466320
Biotin anti-CD19 (MB19-1)	eBioscience	Cat# 13-0191-85, RRID:AB_466385
Biotin anti-IgM (II/41)	eBioscience	Cat# 13-5790-85, RRID:AB_466676
Biotin anti-CD5 (53-7.3)	eBioscience	Cat# 13-0051-85, RRID:AB_466340
APC anti-c-Kit (2B8)	eBioscience	Cat# 17-1171-82, RRID:AB_469430
PE/Cy7 anti-c-Kit (2B8)	eBioscience	Cat# 25-1171-82, RRID:AB_46964
Pacific Blue-c-Kit (2B8)	Biolegend	Cat# 105820, RRID:AB_493476
Alexa Fluor 700 anti-CD34 (RAM34)	eBioscience	Cat# 56-0341-82, RRID:AB_493998
FITC anti-CD34 (RAM34)	eBioscience	Cat# 11-0341-85, RRID:AB_465022
Brilliant Violet 421 anti-CD150 (TC15-12F12.2)	BioLegend	Cat# 115943, RRID:AB_2650881
APC anti-CD150 (TC15-12F12.2)	BioLegend	Cat# 115910, RRID:AB_493460
Brilliant Violet 421 anti-CD41 (MWReg30)	BioLegend	Cat# 133912, RRID:AB_2650893
FITC anti-CD41 (MWReg30)	eBioscience	Cat# 11-0411-85, RRID:AB_763483
PE/Cy7-conjugated anti Ly-6A/E (Sca-1) (D7)	eBioscience	Cat# 25-5981-82, RRID:AB_469669
PerCP/Cy5.5 anti-Ly-6A/E (Sca-1) (D7)	eBioscience	Cat# 45-5981-82, RRID:AB_914372
Alexa Fluor 700 anti-Ly-6A/E (Sca-1) (D7)	eBioscience	Cat# 56-5981-82, RRID:AB_657836
Brilliant Violet 510 anti-Ly-6A/E (Sca-1) (D7)	BioLegend	Cat# 108129, RRID:AB_2561593
PE/Cy7 anti-Ly-6A/E (Sca-1) (D7)	BioLegend	Cat# 122514, RRID:AB_756199
APC/Cy7-CD48 (HM48-1)	BioLegend	Cat# 103432, RRID:AB_2561463
APC anti-CD135 (Flt3) (A2F10)	eBioscience	Cat# 17-1351-82, RRID:AB_10717261
PE anti-CD135 (Flt3) (A2F10)	eBioscience	Cat# 12-1351-83, RRID:AB_465860
PerCP/eFluor 710 anti-CD135 (Flt3) (A2F10)	eBioscience	Cat# 46-1351-82, RRID:AB_10733393
PE/Cy7 anti-CD127 (IL7Ralpha) (A7R34)	BioLegend	Cat# 135014, RRID:AB_1937265
PE anti-CD16/32 (93)	BioLegend	Cat# 101308, RRID:AB_312807
FITC anti-CD16/32 (93)	BioLegend	Cat#101306, RRID:AB_312805
Streptavidin-APC/Cy7	BioLegend	Cat# 405208
Streptavidin-APC/eFluor 780	eBioscience	Cat# 47-4317-82, RRID:AB_10366688
Streptavidin-Brilliant Violet 605	BioLegend	Cat# 405229
PE-Cy7 anti-CD45.1	BioLegend	Cat# 110730, RRID:AB_1134168
Pacific Blue anti-CD45.2	BioLegend	Cat# 109820, RRID:AB_492872
FITC anti-Ly-6G (Gr-1) (RB6-8C5)	eBioscience	Cat# 11-5931-85, RRID:AB_465315)
FITC anti-CD11b (M1/70)	eBioscience	Cat# 11-0112-41, RRID:AB_11042156
APC-eFluor780 CD45R (B220) (RA3-6B2)	eBioscience	Cat# 47-0452-82, RRID:AB_1518810
APC anti-CD3 (17A2)	Biolegend	Cat# 100236, RRID:AB_2561456
APC anti-CD4 (RM4-5)	eBioscience	Cat# 17-0042-83, RRID:AB_469324
APC anti-CD8 (53-6.7)	eBioscience	Cat# 17-0081-83, RRID:AB_469336
APC anti-TER-119 (TER-119)	eBioscience	Cat# 17-5921-83, RRID:AB_469474
eFluor 450 anti-CD41a (MWReg30)	eBioscience	Cat# 48-0411-82, RRID:AB_1582238
FITC anti-CD42a (Xia.B4)	EMFRET Analytics	Cat# M051-1

**Critical Commercial Assays**		

Anti-APC MicroBeads	Miltenyi Biotec	Cat#130-090-855, RRID:AB_244367
LS columns	Miltenyi Biotec	Cat#130-042-401

**Experimental Models: Organisms/Strains**		

Mouse: Female C57BL/6-Ly5.2 NCrSlc	Japan SLC	http://jslc.co.jp/english/index2.htm
Mouse: Male C57BL/6-Ly5.1/5.2-F1	Sankyo-Lab Service	N/A
Mouse: Male Kusabira-Orange transgenic mouse (KuO mouse)	Nakauchi Laboratory at University of Tokyo	https://doi.org/10.1016/j.bbrc.2013.05.017

### Contact for Reagent and Resource Sharing

Further information and requests for resources and reagents should be directed to and will be fulfilled by the Lead Contact, Hiromitsu Nakauchi (nakauchi@stanford.edu).

### Experimental Model and Subject Details

#### Mice

Female C57BL/6-Ly5.2 (Ly5.2) and male C57BL/6-Ly5.1/5.2-F1 (Ly5.1/Ly5.2-F1) mice were purchased from Japan SLC (Shizuoka, Japan) and Sankyo-Lab Service (Tsukuba, Japan), respectively. Eight- to twelve-week-old male Kusabira-Orange mice (KuO mice) served as young donors. Twenty to twenty-four month-old male mice served as aged donors. Eight- to twelve-week-old Ly5.2 female mice served as recipients. All mice were housed in a specific pathogen-free (SPF) condition and were carefully observed by staffs. Animal experiments were approved by the Animal Care and Use Committee, Institute of Medical Science, University of Tokyo.

### Method Details

#### Hematopoietic stem/progenitor cell analysis in untransplanted mice

Bone marrow cells were isolated from tibia, femur and pelvis of male young (8-12 week) and aged (20-24 month) mice and were stained with antibodies as detailed below. Biotin antibody staining was initially performed for 30 minutes, followed by a PBS wash, and a 90-minute stain with the remaining antibodies (all at 4°C). Samples were washed with PBS before analysis. Bone marrow analysis was performed on a FACS AriaII cell sorter (BD Biosciences). Collected data were analyzed with FlowJo software (Tree Star, Ashland, OR). Bone marrow cell type frequencies were calculated from 2x10^6^ live lineage^-^ BM cells per mouse.

Phenotypic cell-surface markers to stain for hematopoietic stem/progenitor cells (including HSCs, MPPs, LMPPs) were stained with a lineage cocktail (biotinylated-CD4, CD8, B220, Gr-1, TER-119 and CD127), FITC-CD34, APC-CD150, Brilliant Violet 421-CD41, PE/Cy7-c-Kit, Brilliant Violet 510-Sca-1, and PE-Flt3 and streptavidin-Brilliant Violet 605.

Phenotypic cell-surface markers to stain for myeloid progenitors (including CMPs, MEPs, GMPs and MkPs) were detected using a lineage cocktail (biotinylated-CD4, -CD8, -B220/CD45RA, -Gr1, -TER-119, -CD127, -CD3, -CD19, and -IgM), FITC-CD34, PE-CD16/32, APC-CD150, Brilliant Violet-CD41, PE/Cy7-c-Kit, Brilliant Violet 510-Sca-1 and streptavidin-APC-Cy7 or -APC/eFluor780.

Phenotypic cell-surface markers to stain for CLP were detected using a lineage cocktail (biotinylated-CD4, -CD8, -B220/ CD45RA, -Gr1, -TER-119, -CD3, and -CD5), PE-Flt3, PE/Cy7-CD127, APC-c-Kit, and Brilliant Violet 510-Sca-1 antibodies, and streptavidin-APC/Cy7 or -APC/eFluor780.

#### Complete blood count analysis

Peripheral blood samples were collected from the retro-orbital venous plexus into capillary tubes filled with powered EDTA. Complete blood count analysis was performed using an automated cell counter (Celltec α, Nihon Koden).

#### Single cell sorting and transplantation

Single cell sorting and transplantation was performed as described previously (Yamamoto et al., 2013). Bone marrow cells were isolated from tibia, femur and pelvis of male young (8-12 week) and aged (20-24 month) KuO mice and were stained for 30 minutes with APC-c-Kit and c-Kit positive cells were enriched using anti-APC magnetic bead (15 minute incubation) and LS columns (Miltenyi Biotec). These cells were then stained for 30 minutes with a lineage cocktail (biotinylated-CD4, -CD8, -B220/CD45RA, -TER-119, -Gr-1, and -CD127). Finally, cells were stained for 90 minutes with Alexa Fluor 700-CD34, Brilliant Violet 421-CD150, FITC-CD41, PE-Cy7-Sca-1 and streptavidin-APC/Cy7 or APC/eFluor 780 and were sorted into 96-well plate with PBS containing 4% FBS on FACS AriaII cell sorted (special order system). For single-cell sorting, the presence of one cell per well was verified under an inverted microscope. Whole bone marrow cells, which served as competitor cells, were isolated from male Ly5.1/Ly5.2-F1 mice and 2 × 10^5^ nucleated cells were transferred into 96-well plate wells. Single KuO cells and competitor cells were transplanted together into lethally irradiated Ly5.2 mice (given two doses of 4.9 Gy, 4 hours apart).

Of the 451 primary recipient mice, 30 died during follow-up, and were eliminated from analyses. Secondary transplantation using cells from femora and tibiae, pelvis, (upper forelimb, and backbone) of the primary recipients were performed to assess self-renewal activity when some myeloid lineages were detected in PB at 24 week after primary transplant. For secondary transplantation, 10^7^ whole BM cells were injected into young female lethally-irradiated (given two doses of 4.9 Gy, 4 hours apart) mice (1-5 per primary recipient).

#### Secondary single cell transplantation

Secondary single cell transplantation using primary recipients that had been transplanted with single cells of young phenotypic HSCs was performed to assess division patterns of transplanted single cell in the primary recipients. At 20 weeks or more after primary transplant, bone marrow cells were isolated from tibia, femur and pelvis, upper forelimb and backbone and were stained with antibodies and sorted using the same protocol as the single cell staining and transplantation section. Single cells of Fractions 1, 2, or 3 were transplanted into young female lethally irradiated mice. Simultaneously, whole bone marrow cells were transplanted into mice (using the same method above) to assess self-renewal activity of the transplanted single cells in the primary recipients.

#### Peripheral blood (PB) analysis

PB was collected from the retro-orbital venous plexus into capillary tubes containing a minimal volume of 10 mM EDTA in water. After erythrocyte lysis with aqueous 140 mM ammonium chloride, cells were stained for 30 minutes with PE/Cy7-CD45.1, Pacific Blue-CD45.2, FITC-Gr-1, FITC-Mac-1, APC/eFluor780-CD45RA/B220, APC-CD3. For analysis of erythrocytes and platelets, one ml of collected blood was stained with APC-TER-119 and eFluor450-CD41. The percentage of chimerism of neutrophils/monocytes, B cells, T cells, erythrocytes, or platelets was defined as the percentage of KuO^+^ cells among CD45.1^+^ B220^-^CD3^-^Gr-1^+^ Mac1^+^ cells, CD45.1^+^Gr-1^-^Mac-1^-^CD3^-^B220^+^ cells, CD45.1^+^Gr-1^-^Mac-1^-^B220^-^CD3^+^ cells, CD41^-^TER-119^+^ cells, or TER-119^-^CD41^+^ cells. The FSC^high^ gate and FSC^low^ gate were used for analysis of erythrocytes and platelets, respectively. PB analysis was performed on a Gallios (Beckman Coulter, Fullerton, CA, USA), FACSCanto, or FACS AriaII cell sorter (BD Biosciences). Collected data were analyzed with FlowJo software (Tree Star, Ashland, OR). Recipient mice were defined as reconstituted when at least one type of mature blood lineage contained donor-derived KuO^+^ cells at 0.005% or more, to avoid overlooking reconstitution especially of platelets and erythrocytes. PB chimersim was calculated based on 30,000-50,000 leukocytes, 20,000-50,000 platelets, and 500,000 erythrocytes, per mouse/time point.

#### Hematopoietic stem/progenitor cell staining for frequency analysis in transplanted mice

We analyzed BM cells of primary and secondary recipients to determine the frequency of KuO^+^ phenotypic stem and progenitor cell population. Whole bone marrow cells were isolated from tibia, femur, pelvis, (upper forelimb bones, and backbones) of recipient mice and were stained with antibodies, as detailed below. Biotin antibody staining was initially performed for 30 minutes, followed by a PBS wash, and a 90-minute stain with the remaining antibodies (all at 4°C). Samples were washed with PBS before analysis. Bone marrow analysis was performed on a Gallios (Beckman Coulter, Fullerton, CA, USA), FACSCanto, or FACS AriaII cell sorter (BD Biosciences). Collected data were analyzed with FlowJo software (Tree Star, Ashland, OR). Bone marrow cell type frequencies were calculated from 2x10^6^ live lineage^-^ BM cells per mouse.

Phenotypic cell-surface markers to stain for hematopoietic stem/progenitor cells (including HSCs, MPPs, LMPPs) were stained with a lineage cocktail (biotinylated-CD4, CD8, B220, Gr-1, TER-119 and CD127), Alexa Fluor 700-CD34, Brilliant Violet 421-CD150, FITC-CD41, PE/Cy7-c-Kit, PerCP/Cy5.5-Sca-1, and APC-Flt3 and streptavidin-APC-Cy7 or -APC/eFluor780; or Alexa Fluor 700-CD34, PE/Cy7-CD150, Pacific Blue-CD41, PE/Cy7-c-Kit, Brilliant Violet 510-Sca-1, and PerCP/eFluor 710-Flt3 and streptavidin-APC-Cy7 or -APC/eFluor780.

Phenotypic cell-surface markers to stain for myeloid progenitors (including CMPs, MEPs, GMPs and MkPs) were detected using a lineage cocktail (biotinylated-CD4, -CD8, -B220/CD45RA, -Gr1, -TER-119, -CD127, -CD3, -CD19, and –IgM), Alexa Fluor 700-CD34, FITC-CD16/32, PE/Cy7-CD150, Brilliant Violet 410- or eFluor 450-CD41, APC-c-Kit, Brilliant Violet 510-Sca-1 antibodies, and streptavidin-APC-Cy7 or -APC/eFluor780.

Phenotypic cell-surface markers to stain for CLP were detected using a lineage cocktail (biotinylated-CD4, -CD8, -B220/ CD45RA, -Gr1, -TER-119, -CD3, and -CD5), APC-Flt3, PE/Cy7-CD127, Pacific Blue-c-Kit, and Alexa Fluor 700-Sca-1 antibodies, and streptavidin-APC/Cy7 or -APC/eFluor780; or APC-Flt3, PE/Cy7-CD127, Pacific Blue-c-Kit, and Alexa Fluor 700-Sca-1 antibodies, and streptavidin-APC/Cy7 or -APC/eFluor780 or PerCP/eFluor 710-Flt3, APC/Cy7-CD127, Pacific Blue-c-Kit, and Alexa Fluor 700-Sca-1 antibodies, and streptavidin-APC/Cy7 or -APC/eFluor780; or APC-Flt3, PE/Cy7-CD127, PE/Cy7-c-Kit, and Brilliant Violet 510-Sca-1, and streptavidin-Brilliant Violet 605.

### Quantification and Statistical Analyses

Chimerism comparisons were performed using unpaired t test (Figure 2C). Statistical analysis in Figures S1B and S1C were performed with Mann-Whitney U-test (two-tail). Statistical analysis was performed using chi-square test (Figure S1G). Multiple comparison in each of young LT-HSCs, aged LT-HSCs and latent HSCs were calculated using SNK test (Figure S3C).

### Data and Software Availability

The software for data analysis included Flowjo, GraphPad Prism, and Microsoft Excel.

## Supplemental Information

Document S1. Figures S1–S4 and Tables S1–S3

Document S2. Article plus Supplemental Information
