# Supplementary material for: Large-Scale Clonal Analysis Resolves Aging of the Mouse Hematopoietic Stem Cell Compartment
Source: Cell Stem Cell. 2018 Apr 5;22(4):600–607.e4. doi: 10.1016/j.stem.2018.03.013 (PMC5896201; doi:10.1016/j.stem.2018.03.013)
Supplement: Document S1. Figures S1–S4 and Tables S1–S3 [file mmc1.pdf]

Cell Stem Cell, Volume *22*

Supplemental Information

Large-Scale Clonal Analysis Resolves Aging

# of the Mouse Hematopoietic Stem Cell Compartment

Ryo Yamamoto, Adam C. Wilkinson, Jun Ooehara, Xun Lan, Chen-Yi Lai, Yusuke Nakauchi, Jonathan K. Pritchard, and Hiromitsu Nakauchi

Supplementary Figure S1

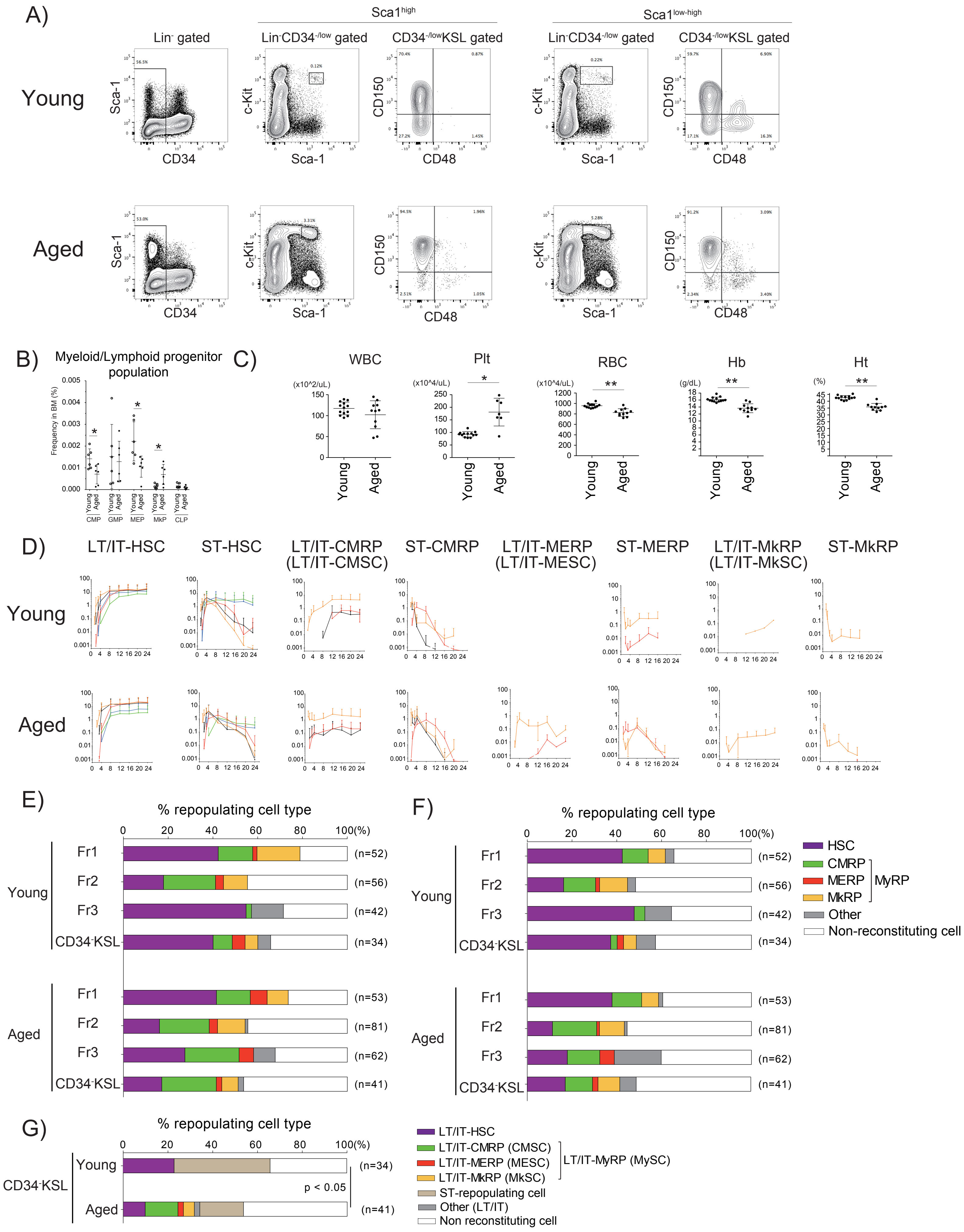

Supplementary Figure S2

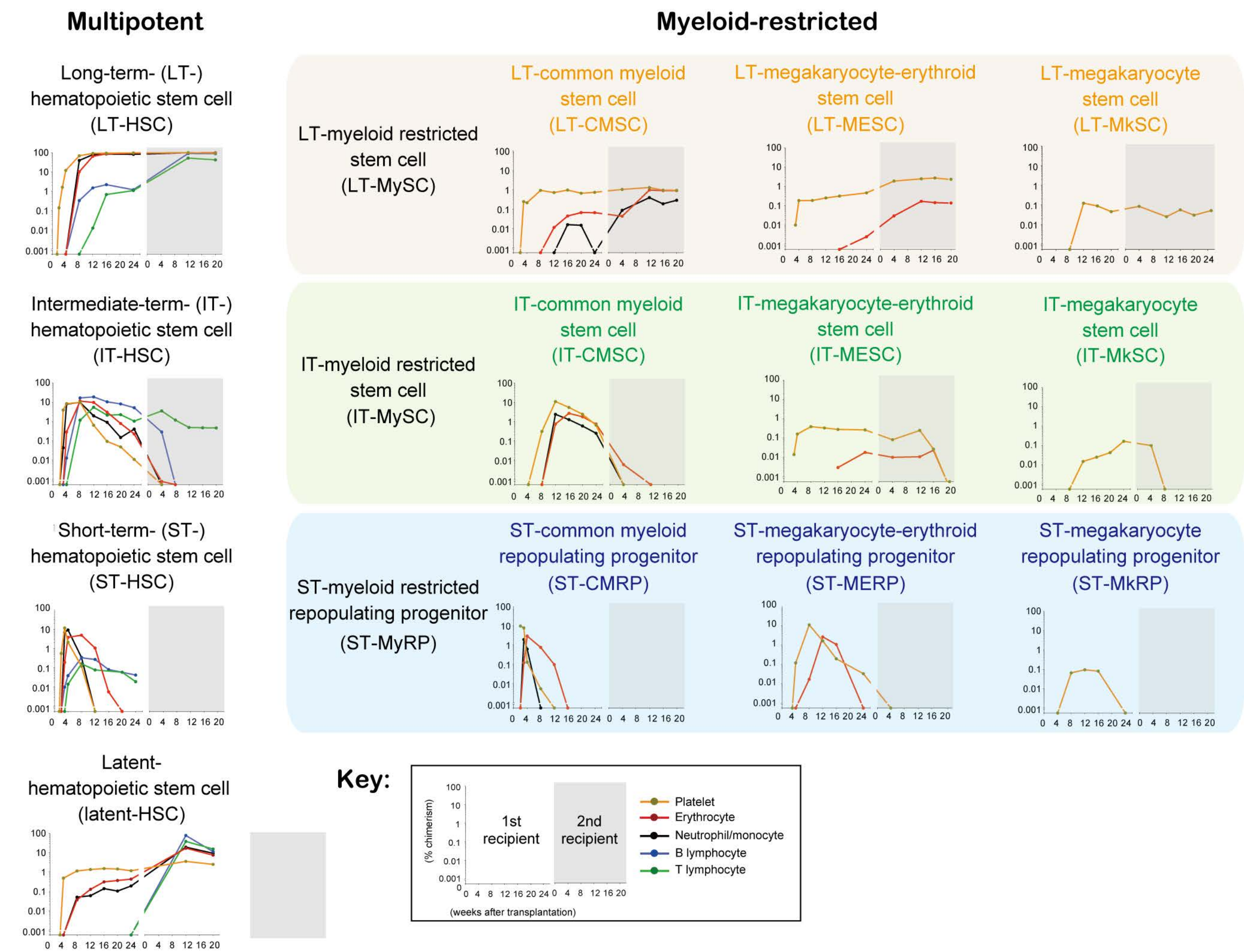

Supplementary Figure S3

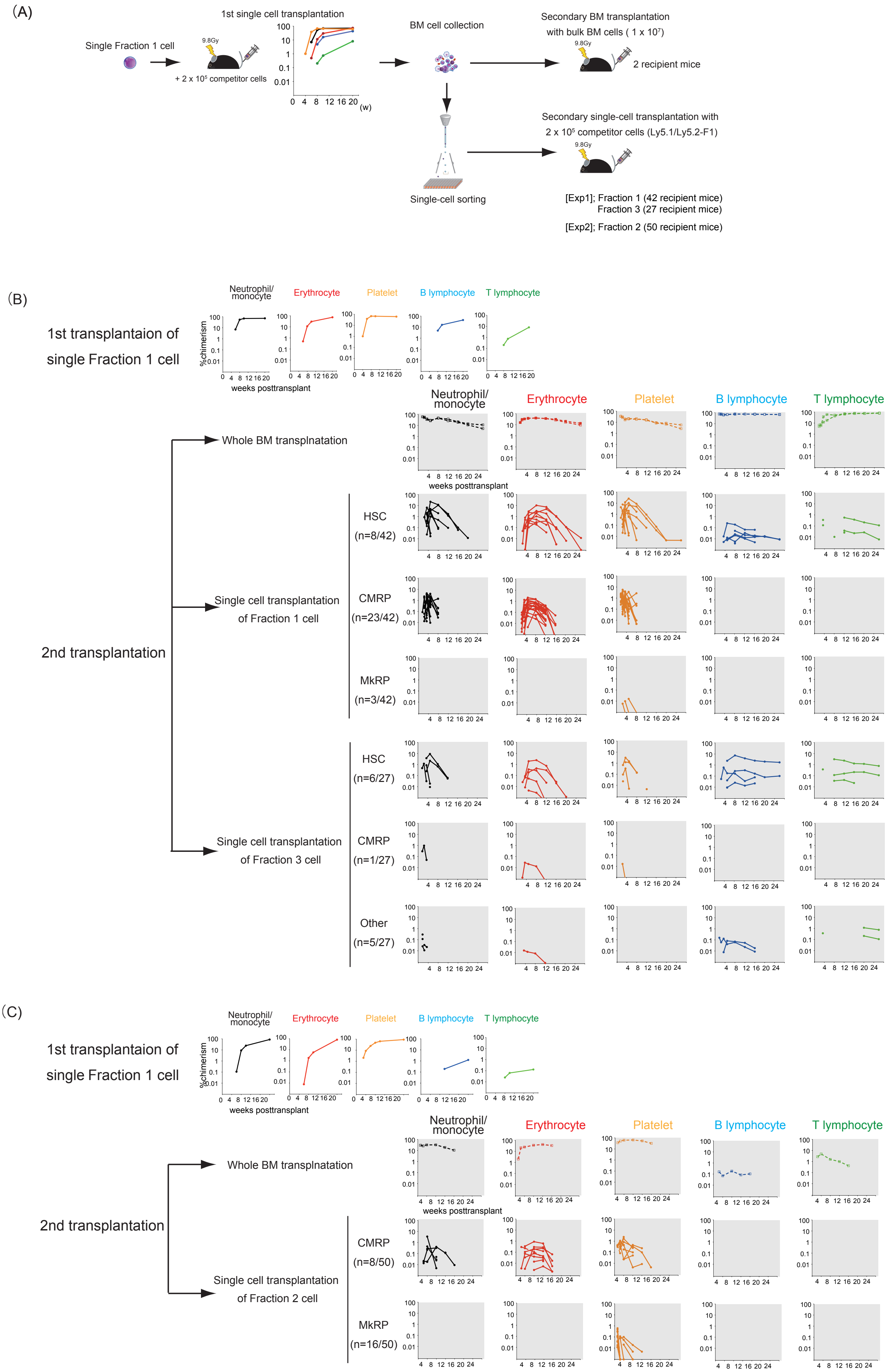

Supplementary Figure S4

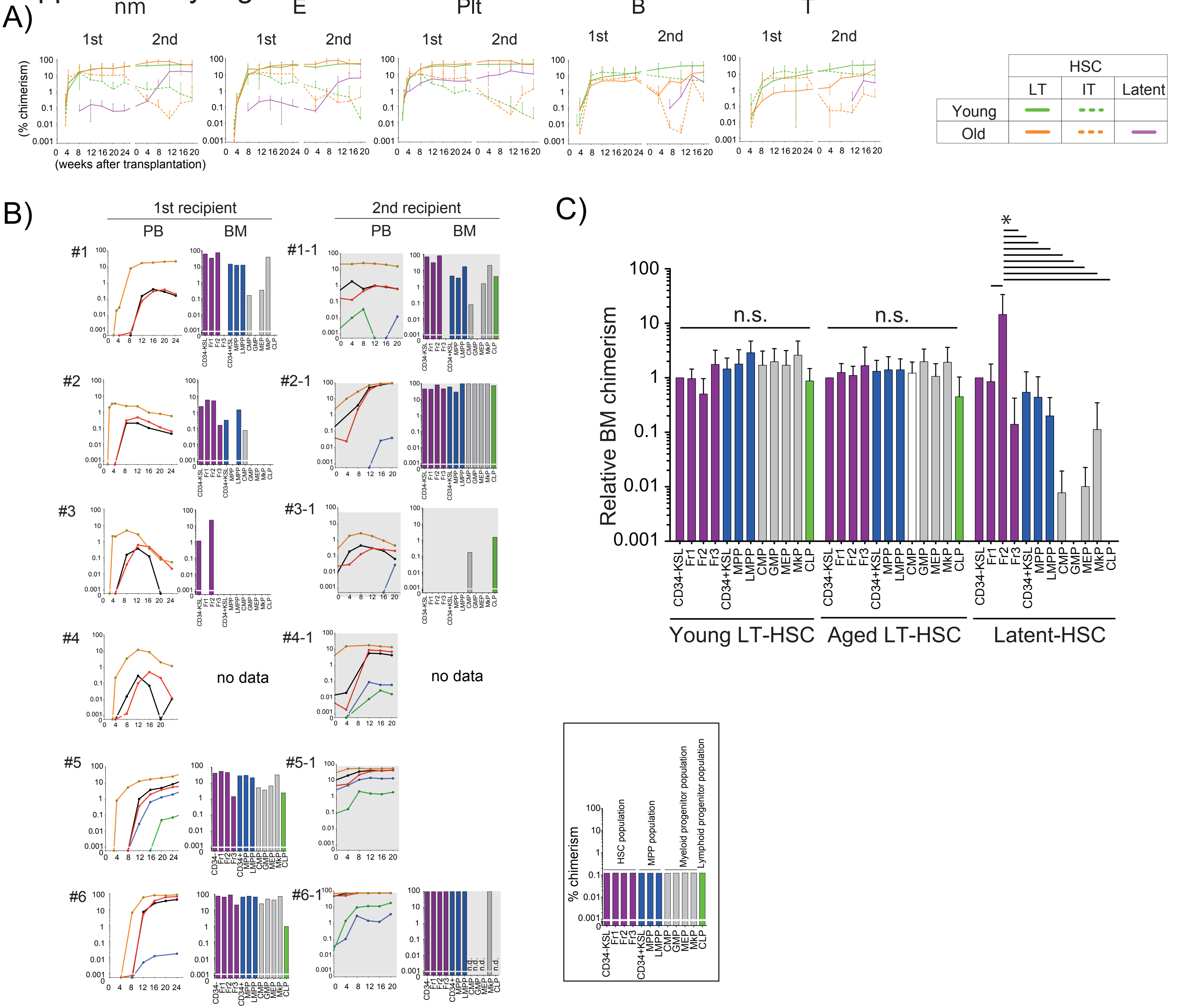

Supplementary Information relating to:

Large-scale clonal analysis resolves aging of the mouse hematopoietic stem cell compartment by Ryo Yamamoto, Adam C. Wilkinson, Jun Ooehara, Xun Lan, Chen-Yi Lai, Yusuke Nakauchi, Jonathan K. Pritchard, Hiromitsu Nakauchi

Contents:

Supplementary Table S1, related to Table 1

Supplementary Table S2, related to Figure 1

Supplementary Table S3, related to Figure 2

Supplementary Figure S1, related to Figure 1

Supplementary Figure S2, related to Figure 2

Supplementary Figure S3, related to Table 1

Supplementary Figure S4, related to Figure 3

Table S1. Related to Table 1: Estimated frequencies of functional cell types within the phenotypic HSC compartment

Functional cell type	Frequency in $Vwf^{+}CD150^{+}CD48^{-}KSL$*	Functional cell type	Frequency in young $CD150^{+}CD34^{-}KSL$ (Fraction 1 + Fraction 2)
**P-HSC***	4.1 %	**LT/IT-MkRP**	2.6 %
**PE-HSC***	1.0 %	**LT/IT-MERP**	1.4 %
**PEM-HSC***	6.1 %	**LT/IT-CMRP**	4.5 %
**PEMB/PEMBT-HSC***	26.0 %	**LT/IT-HSC**	28.3 %

Frequency in young $CD150^{+}CD34^{-}KSL$ (Fraction 1 + Fraction 2) was derived from **Table 1**. P-, PE-, PEM-, and PEMB/PEMBT-HSCs reported by Carrelha correspond to LT/IT-MkRP, LT/IT-MERP, LT/IT-CMRP, and LT/IT-HSCs using our definitions. Abbreviations: platelet-hematopoietic stem cell, P-HSC; platelet/erythroid-hematopoietic stem cell, PE-HSC; platelet/erythroid/myeloid-hematopoietic stem cell, PEM-HSC; platelet/erythroid/ myeloid /B-lymphocyte-hematopoietic stem cell, PEMB-HSC; and platelet/erythroid/ myeloid /B lymphocyte/T lymphocyte-hematopoietic stem cell, PEMBT-HSC. *Data from Carrelha et al., 2018. $Vwf^{+}CD150^{+}CD48^{-}KSL$ cells are estimated to be 38% of $CD150^{+}CD34^{-}KSL$ population.

Table S2. Related to Figure 1: Estimated frequencies of each functional cell type among nucleated bone marrow cells, based on primary transplantation assay results

	Young	Aged	Fold increase
**HSC**	23.7	123.0	5.2
**MyRP/MySC**	21.2	275.0	13.0
CMRP/CMSC	*11.3*	*160.0*	*14.2*
MERP/MESC	*2.0*	*28.2*	*14.1*
MkRP/MkSC	*7.9*	*86.2*	*10.9*
**Others**	2.3	10.8	4.7
**Non-reconstituting**	20.3	317.0	15.6

The estimated number of each cell type per 10^6 BM cells and fold increase from young to aged BM, calculated to one decimal place. Frequencies of each cell type were estimated using results of single cell transplantation assays and frequencies of Fraction 1, Fraction 2, and Fraction 3. Abbreviations: myeloid-restricted repopulation progenitor/myeloid-restricted stem cell, MyRP/MySC; common myeloid repopulation progenitor/common myeloid stem cell, CMRP/CMSC; megakaryocyte-erythroid repopulation progenitor/megakaryocyte-erythroid stem cell, MERP/MESC; and megakaryocyte repopulation progenitor/megakaryocyte stem cell, MkRP/MkSC. CMRP/CMSC, MERP/MESC and MkRP/MkSC are included in MyRP/MySC.

Table S3. Related to Figure 2: Frequencies of each cell type among nucleated bone marrow cells from secondary transplant assay

Functional cell type	Reconstitution duration	Young	Aged	Fold increase
HSC	Long-term (LT)	7.3	21.0	2.9
HSC	Intermediate-term (IT)	5.6	49.9	9.0
HSC	Short-term (ST)	10.8	52.4	4.9
MySC	Long-term (LT)	0.7	0.0	n/a
MySC	Intermediate-term (IT)	3.3	63.8	19.6
MyRP	Short-term (ST)	17.2	129.0	7.5
Latent-HSC	Long-term (LT)	0.0	82.0	n/a

The estimated number of each cell type per 10^6 BM cells and fold increase from young to aged BM, calculated to one decimal place. Frequencies of each cell type were estimated using results of single cell transplantation assays and frequencies of Fraction 1, Fraction 2, and Fraction 3. Abbreviations: myeloid-restricted stem cell, MySC; and myeloid-restricted repopulation progenitor, MyRP. MyRP includes CMRP, MERP, and MkRP. MySC includes CMSC, MESC and MkSC.

Supplementary Figure Legends

Figure S1. Related to Figure 1: Gating strategy for sorting HSCs for single cell transplantation and functional comparison of young and aged HSCs by single cell transplantation

(A) Gating strategies for isolation of young and aged pHSCs comparing Sca-1^{high} and Sca-$1^{low\text{-}high}$ gating strategies to define KSL population.

(B) Frequency of myeloid/lymphoid progenitor population in the bone marrow (BM). Percentage of $CD41^{-}CD34^{+}CD16/32^{-}KS^{-}L$ (referred to as CMP), $CD41^{-}CD34^{+}CD16/32^{+}KS^{-}L$ (referred to as GMP), $CD41^{-}CD34^{-}CD16/32^{-}KS^{-}L$ (referred to as MEP), $CD150^{+}CD41^{+}KS^{-}L$ (referred to as MkP), $IL7Ra^{+}Flt3^{+}K^{mid}S^{mid}L$ (referred to as CLP) in whole BM of six individual young and aged mice are shown. * denotes $p < 0.05$.

(C) Complete blood count of peripheral blood (PB) in young and aged mice. PB was collected from thirteen young and eleven aged mice and analyzed for complete blood count. WBC: white blood cell; RBC: red blood cells; HCT: hematocrit; Hb: hemoglobin. * and ** denote $p < 0.05$ and $p < 0.01$, respectively.

(D) Average chimerism kinetics of young and aged HSCs (LT/IT and ST), CMRPs (LT/IT and ST), MERP (LT/IT and ST) and MkRP (LT/IT and ST) within nm, E, P, B, and T lineages.

(E) Frequency of HSCs, CMRPs, MERPs, MkRPs and "other" are shown in mice transplanted with young or aged Fraction 1, Fraction 2, Fraction 3, and $CD34^{-}KSL$ cells, as defined by single cell transplantation assays using chimerism threshold 0.005%. "Non-reconstituting" denotes no peripheral blood reconstitution of KuO^{+} cells in primary recipients. CMRPs, MERPs and MkRPs are subsets of MyRPs.

(F) The same analysis as Figure S1D using chimerism threshold 0.1%.

(G) Frequency of each cell type (HSCs, CMRPs/CMSCs, MERPs/MESCs, MkRPs/MkSCs and "other") with long-term (LT) or intermediate-term (IT) and short-term (ST) repopulating cells are displayed for young or aged $CD34^{-}KSL$ cells. Statistical analysis comparing LT/IT-HSC, LT/IT-MyRP (MySC), other, ST-RC, and non-reconstituting cells was performed using

chi-square test.

Figure S2. Related to Figure 2: Glossary of functional cell types within the mouse CD34⁻KSL pHSC population

A summary of the terminology used to describe the various functional cell types that make up the young and aged pHSC compartment and representative PB lineage output following primary and secondary transplantation (see figure key for details).

Figure S3. Related to Table 1: Single cell secondary transplantation of young LT-HSC

(A) Schematic of experimental approached used in primary and secondary single cell transplantation experiments. Single young $CD150^{+}CD41^{-}CD34^{-}$KSL cell (Fraction 1) were transplanted into young mice with $2x10^{5}$ competitor cells. Whole BM cell ($1x10^{7}$) secondary transplantation assays as well as single cell secondary transplantation assays were performed 20 weeks after primary transplantation. Secondary single cell transplantations using Fraction 1 and Fraction 3 cells (**C**) and Fraction 2 cells (**D**) were performed in two independent experiments. PB chimerism was periodically checked over 24 weeks.

(B) Primary recipient PB chimerism displayed above and secondary recipient PB chimerism below. Secondary single cell transplantation of Fraction 1 and Fraction 3 cells was performed with competitor cells into 42 and 27 mice, respectively. Whole BM cells were transplanted into two mice. Secondary transplanted single cells from Fractions 1 and 3 showed HSC (19%), CMRP (54.8%), MkRP (7.1%) and no reconstitution (20%), and HSC (22.2%), CMRP (3.7%), others (18.5%), and no reconstitution (55.6%).

(C) Primary recipient PB chimerism displayed above and secondary recipient PB chimerism below. Secondary single cell transplantation from Fraction 2 were simultaneously performed with competitor cells into 50 mice. Whole BM cells were transplanted into two mice. Secondary transplanted single cells from Fraction 2 showed CMRP (16%), MkRP (32%), and no reconstitution (52%).

Figure S4. Related to Figure 3: Primary and secondary transplantation of young and aged pHSCs

(A) Average chimerism kinetics of nm, E, P, B, and T are shown in young and aged LT- and IT-HSCs, and latent-HSCs.

(B) The frequency of KuO^+ phenotypic stem and progenitor cells within the BM from a representative subset of primary recipient mice and secondary recipient mice (24 weeks after primary transplantation and 20 weeks after secondary transplantation) containing latent-HSCs (#1-#4), young LT-HSC (#5) and aged LT-HSC (#6). Chimerism of KuO^+ phenotypic HSCs, Fraction 1, Fraction 2, Fraction 3 (highlighted in purple), MPPs, LMPPs (highlighted in blue), CMPs, GMPs, MEPs, MkPs (highlighted in grey), or CLPs (highlighted in green) are show in the bar graph. n.d. denotes no data.

(**C**) Average fold-change in the frequency of KuO^+ phenotypic BM stem and progenitor populations in primary recipients transplanted with single functionally-defined young LT-HSCs (n=12), aged LT-HSCs (n=5), and aged latent-HSCs (n=7) at 24-weeks post-transplantation. Chimerism displayed relative to the chimerism within the total $CD34^-$KSL compartment. Multiple comparison in each of young LT-HSCs, aged LT-HSCs, and latent HSCs were calculated using SNK test. * denotes $p < 0.01$. As latent-HSCs were a Fraction 2 cells in donor mice, comparison performed against Fraction 2 in primary recipient.
